# Deoiledjatropha seed cake is a useful nutrient for pullulan production

**DOI:** 10.1186/1475-2859-11-39

**Published:** 2012-03-30

**Authors:** Anirban Roy Choudhury, Nishat Sharma, GS Prasad

**Affiliations:** 1 Biochemical Engineering Research & Process Development Centre (BERPDC), CSIR-Institute of Microbial Technology (IMTECH), Council of Scientific and Industrial Research (CSIR), Sector - 39A, Chandigarh 160 036, India; 2 Biochemical Engineering Research & Process Development Centre (BERPDC), CSIR-Institute of Microbial Technology (IMTECH), Council of Scientific and Industrial Research (CSIR), Sector - 39A, Chandigarh 160 036, India; 3 Microbial Type Culture Collection and Gene Bank (MTCC), CSIR-Institute of Microbial Technology (IMTECH), Council of Scientific and Industrial Research (CSIR), Sector - 39A, Chandigarh 160 036, India

**Keywords:** Jatropha, Value addition to waste, *Aureobasidium pullulans*, Fermentation, Exopolysaccharide, Pullulan

## Abstract

**Background:**

Ever increasing demand for fossil fuels is a major factor for rapid depletion of these non-renewable energy resources, which has enhanced the interest of finding out alternative sources of energy. In recent years jatropha seed oil has been used extensively for production of bio-diesel and has shown significant potential to replace petroleum fuels at least partially. De-oiled jatropha seed cake (DOJSC) which comprises of approximately 55 to 65% of the biomass is a byproduct of bio-diesel industry. DOJSC contains toxic components like phorbol esters which restricts its utilization as animal feed. Thus along with the enhancement of biodiesel production from jatropha, there is an associated problem of handling this toxic byproduct. Utilization of DOJSC as a feed stock for production of biochemicals may be an attractive solution to the problem.

Pullulan is an industrially important polysaccharide with several potential applications in food, pharmaceuticals and cosmetic industries. However, the major bottleneck for commercial utilization of pullulan is its high cost. A cost effective process for pullulan production may be developed using DOJSC as sole nutrient source which will in turn also help in utilization of the byproduct of bio-diesel industry.

**Results:**

In the present study, DOJSC has been used as a nutrient for production of pullulan, in place of conventional nutrients like yeast extract and peptone. Process optimization was done in shake flasks, and under optimized conditions (8% DOJSC, 15% dextrose, 28°C temperature, 200 rpm, 5% inoculum, 6.0 pH) 83.98 g/L pullulan was obtained. The process was further validated in a 5 L laboratory scale fermenter.

**Conclusion:**

This is the first report of using DOJSC as nutrient for production of an exopolysaccharide. Successful use of DOJSC as nutrient will help in finding significant application of this toxic byproduct of biodiesel industry. This in turn also have a significant impact on cost reduction and may lead to development of a cost effective green technology for pullulan production.

## Background

Fossil fuels especially, fuels and commodities obtained from petroleum derived liquids play an important part in almost every aspect of our modern life. However, over exploitation of these natural resources to maintain modern amenities has caused negative ramifications on environmental as well as economical aspects of our life. Due to the limited nature of fossil fuels, their prices are expected to increase more rapidly in the near future [1]. Therefore, to reduce the use of fossil fuels there is a compelling need to search alternative sources of energy. Production of fuels and chemicals from renewable biomass is becoming increasingly attractive and will be essential for our future and sustainability [2]. In recent years, *Jatropha curcas *L. has gained considerable attention as a potential source of biodiesel and many plantations of jatropha have been established in tropical and subtropical regions worldwide [3,4]. SWOT (Strength, Weakness, Opportunity and Threat) analysis carried out on the feasibility of jatropha biofuels suggests that to make it economically viable it is important to utilize by products produced during the biodiesel production from jatropha. De-oiled jatropha seed cake (DOJSC) produced during oil extraction is the major by product (55%-65% biomass) and contains several toxic substances which include phorbol esters, curicine etc. [5]. Presence of these toxic substances make de-oiled jatropha seed cake unsuitable as a feed for animal and poultry. Thus along with the development of biodiesel production from jatropha, there exists an inherent problem of handling this byproduct. Therefore, it is also important to find out suitable uses for the jatropha seed cake to sort out these problems. Studies have shown that this de-oiled jatropha seed cake is rich in different nutrients like minerals, amino acids etc. [6] suggesting that it may be possible to use this as a nutrient for production of valuable products.

Pullulan is an industrially important biopolymer having wide range of applications in food, pharma and cosmetic industries. In spite of several published reports on pullulan production via fermentation [7-9], cost of pullulan is high compared to other biopolymers like xanthan gum etc. [10]. Therefore, it is important to develop a suitable fermentation process which can make the product economical, either by increasing the yield or by lowering the cost of the media components. In most of the earlier published reports efforts were made to enhance the yield of pullulan production [7-9,11]. However, it is important to note that media components add significant cost to the production, and it may even reach up to 30% of the total production cost [12]. In some of the earlier reports urea, ammonium salts etc. as an alternative nitrogen source for pullulan production [13,14]. However, the yields reported in these cases were not good enough to make the process economical. Therefore, utilization of industrial by products for production of pullulan with desired yield may make the process economically viable.

Except a report describing utilization of soyabean pomace as nitrogen source for production of pullulan, in which the yield was very low [15], there are no other significant reports where agricultural residues were used as nutrient for production of pullulan. Recently, we have reported high pullulan production (66.79 g/L) by an osmotolerant yeast *Aureobasidium pullulans *RBF 4A3 using glucose, yeast extract and peptone as nutrients[8]. In the present study we have examined potential of de-oiled jatropha seed cake (DOJSC) as a nutrient to substitute costly nutrients like yeast extract and peptone for production of pullulan by *Aureobasidium pullulans *RBF-4A3. The process of pullulan production was optimized in shake flask and the overall pullulan production and yield obtained is high compared to earlier published reports [16,17]. This is the first report of pullulan production using de-oiled jatropha seed cake as nutrient. A process economic analysis has shown that use of DOJSC as nutrient can reduce the raw material cost considerably and this may lead to development of successful commercial process for pullulan production. Therefore, the outcome of the present study will not only help in waste minimization and value addition to the waste produced during bio-diesel production from jatropha, but also help in reduction of raw material cost for pullulan production.

## Results and discussions

### Optimization of culture conditions

The pullulan production process was optimized using single point optimization technique. Influences of different factors like DOJSC concentration in media, incubation temperature, agitation speed, inoculum size and initial pH of the media on pullulan production were examined.

### Effect of concentration of de-oiled jatropha seed cake in production media

**Figure 1:**
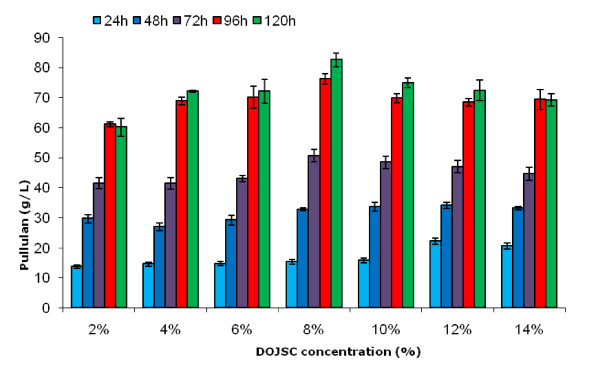


DOJSC is a rich source of protein (56.4-63.8%) and also contain fibers (8.1-9.1%) [18]. Although DOJSC is very rich in essential nutrients especially amino acids for growth of microbes, at higher concentrations it may be detrimental for growth and product formation. Hence it is important to find out optimum concentration of DOJSC for pullulan production. Shake flask fermentations were carried out by varying DOJSC concentration from 2% to 14% (w/v) in the production media. Initially the pullulan production increased with increase in DOJSC concentration from 61.18 g/L at 2% (w/v) to 82.57 g/L at 8% (w/v) concentration. However at concentration beyond 8% gradual reduction in pullulan production was observed and it went down to 69.21 g/L when DOJSC was increased up to 14% DOJSC (Figure 1). This may be attributed to that fact that higher concentration of DOJSC has a negative impact on the metabolic activities of microbial cells, which ultimately affect the production of the exopolysaccharide.

### Effect of incubation temperature on pullulan production

**Figure 2:**
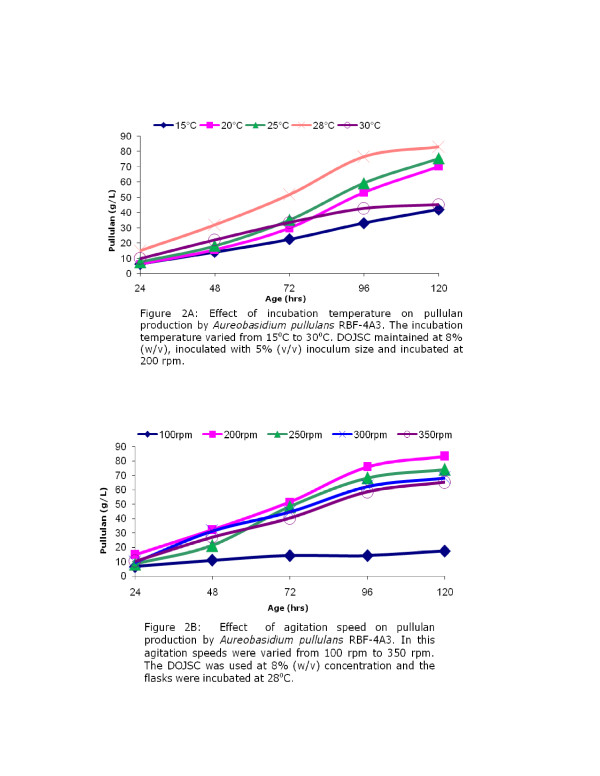


Incubation temperature is strain dependent and has significant influence on growth and production of pullulan during fermentation [19] suggesting the need to determine optimum temperature to maximize pullulan production. The incubation temperature was varied from 15°C to 30°C to examine the effect of the same on pullulan production. Optimum pullulan production was obtained at 28°C (82.91 g/L) and it was almost twice as compared to the production at 15°C (Figure 2A). However, as the temperature was increased to 30°C there was significant reduction in pullulan production (45.11 g/L). This observation is in agreement with the observations of Roukas and Biliaderis [20], but different from other report which shows optimal pullulan production at 25°C [21]. This confirms the earlier observations that the temperature optimum for production of pullulan is strain specific and also often corresponds to the optimum temperature of growth of the microorganism used for production.

### Effect of agitation speed on pullulan production

Agitation speed determines the level of mixing and homogeneity during fermentation and helps in maintenance of a concentration gradient between exterior and interior of the cells by continuous surface renewals. A steady concentration gradient ensures smooth and continuous transport of substrates, other nutrients and products across the cell wall. However, high shear is generated at higher agitation speeds may lead to cell damage and thus affect the growth and polymer production. Therefore, it is important to find out optimum condition of agitation to achieve higher level of production. In the present study, effect of agitation speed was examined at 5 different levels (100, 200, 250, 300, 350 rpm). The results obtained clearly shows that agitation speed of 100 supported very little (17.37 g/L) pullulan production, indicating that it was too low for production. All other agitation speeds supported almost similar pullulan production up to 72 hours, after which 200 rpm was found to be the best for pullulan production (83.13 g/L) with increase in rpm beyond 200, there was a gradual decrease in pullulan production (Figure 2B).

### Effect of inoculum size on pullulan production

**Figure 3:**
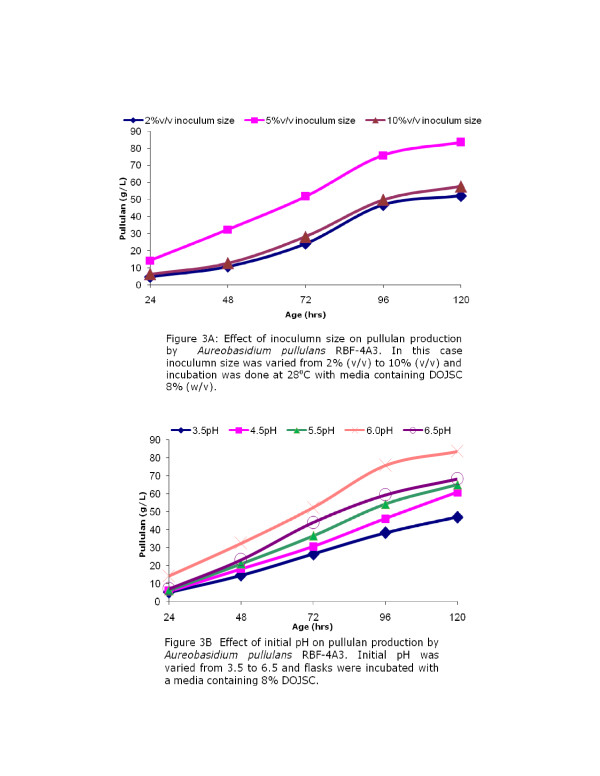


Inoculum size has significant effect on the productivity in fermentation processes as it defines the initial microbial load in the fermentation system and thus controls duration of lag phase and subsequently production of the target metabolites. It has been observed that inoculum size has significant effect on the cell morphology and growth pattern of *Aureobasidium pullulans*, and change in morphology has significant effect on pullulan production [22]. In order to find out optimum inoculum size for pullulan production, its concentration was varied from 2% to 10% (v/v). The results obtained show that pullulan production is low when 2% (v/v) inoculum was used and increased to 83.46 g/L pullulan was obtained at the end of 120 hours fermentation when 5% (v/v) inoculum was used. Further increase of inoculum size up to 10% adversely affected pullulan production (Figure 3A) suggesting that 5%(v/v) concentration is optimal for pullulan production.

### Effect of initial pH on pullulan production

pH plays and important role in metabolic activities of microbial cells and it has been observed that cell morphology of *Aureobasidium pullulans *depends on the pH of the media which in turns affects the production of the polymer [23,24]. In present study the initial pH of the media was varied from 3.5 to 6.5 to understand its effect on pullulan production. Pullulan production was less at an initial pH of 3.5 and increases with increase in initial pH up to 6.0 (Figure 3B) and decreases at higher pH (6.5). It was observed that an initial pH of 6.0 is most suitable for the production of pullulan (83.59 g/L). In earlier reports Roukas and Biliaderis [25] observed that maximum pullulan production at a pH of 6.5, whereas Auer and Seviour [26] reported maximum pullulan production at an initial pH of 7.5. The results obtained along with earlier published reports clearly indicate that the pH optima depend on the strain as well as on the substrate used for production of polymer.

### Validation of shake flask experiments in a laboratory scale fermenter

**Figure 4:**
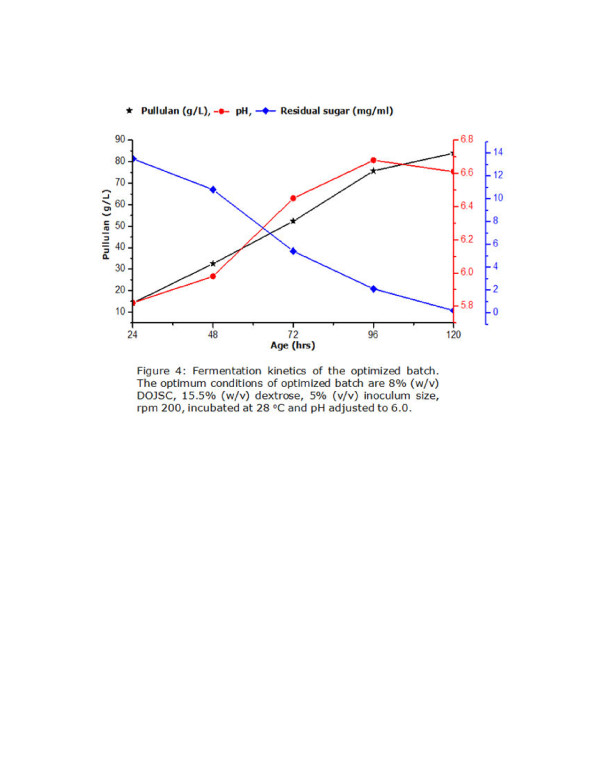


The optimized batch was run in a 5 L fermenter (New Brunswick Scientific, Bioflow 310) with 3.5 L working volume to validate the observations of shake flask experiments. The fermentation kinetics of the optimized batch (Figure 4) showed that the rate of polysaccharide production is directly proportional with consumption of carbon source. The pH of the medium increased during the process till 96 hours and 83.98 g/L of pullulan was produced at the end of the batch. FT-IR data (Additional file 1: Figure S1) shows that the spectra obtained for standard pullulan (Sigma) and the pullulan produced using DOJSC as a nutrient are almost identical confirming the chemical structure of the polymer obtained via fermentation with DOJSC as nutrient is same as standard sigma pullulan. The overall results obtained indicate that the process optimized in the shake flask level may be scaled up easily to fermenter and it may lead to development of a successful and cost effective technology for pullulan production. It should also be noted that optimum production obtained in fermenter is significantly high as compared to earlier reports (Table 1). The yield obtained here is also significantly high as compared to earlier reports published and hence, may have significant impact on the production cost of the polymer.

**Table 1:** Pullulan production using different substrates as carbon and nitrogen source

Name of microorganism	Substrates		Fermentation Mode	Fermentation Volume	Pullulan production (g/L)	Productivity of Pullulan (g/L.h)	Yields of Pullulan	References
	**Carbon source**	**Nitrogen source**					**g** **pullulan/g** **sugar consumed)**	

*Aureobasidium pullulans*	Glucose	Yeast extract, Peptone	Batch	50 ml	66.8	0.7	0.5	[8]

*Aureobasidium pullulans*	Glucose	Soyabean pomace	Batch	100 ml	7.6	0.1	0.4	[15]

*Aureobasidium pullulans*	Sucrose	Beet molasses	Batch	100 ml	32	0.3	0.5	[27]

*Aureobasidium pullulans*	Jaggery	Yeast extract	Batch	50 ml	51.9	0.7	0.5	[28]

*Aureobasidium pullulans*	Glucose	Ammonium sulphate	Continuous	800 ml	3.6	0.4	0.4	[29]

*Aureobasidium pullulans*	Sucrose	Ammonium sulphate	Continuous	1.5 L	23.1	0.9	0.6	[30]

*Aureobasidium pullulans*	Sucrose	Beet molasses	Batch	5 L	35	0.3	0.7	[31]

*Aureobasidium pullulans*	Glucose	DOJSC	Batch	3.5 L	83.9	0.7	0.7	Current study

### Process economics

Cost analysis of ingredients used in fermentation process for production showed that major cost of the production of the polymer is associated with the cost of nitrogen sources used. DOJSC contains around 56-63% protein and 8-9% fiber, and therefore, it can be used as a nutrient for replacing conventional nitrogen sources. As, the cost of DOJSC is much less as compared to conventional nitrogen sources, replacing the conventional nitrogen sources with DOJSC results in significant cost reduction. A process economics analysis suggest that the use of de-oiled jatropha seed oil cake can reduce the raw material cost by 94% as compared to conventional nitrogen sources like yeast extract and peptone (Table 2). This may be helpful in economical production of pullulan along with utilization of the DOJSC.

**Table 2:** Process Economic analysis for raw material cost calculation for production of pullulan using DOJSC

Raw material	Process I					Process II				
	**Requirement (g/L media)**	**Rate ($/Kg)**	**Cost/L media** **($)**	**Pullulan produced** **g/L**	**Cost of raw material/Kg** **Pullulan produced** **($)**	**Requirement** **(g/L media)**	**Rate** **($/Kg)**	**Cost/L media** **($)**	**Pullulan produced** **(g/L)**	**Cost of raw material/Kg Pullulan** **produced ($)**

Glucose	150	0.8	0.1	66.8	1.8	150	0.8	0.1	83.13	1.4

Yeast Extract	15	70	1.1		15.7	-				
				
Peptone	20	28	0.6		8.4	-				
				
DOJSC	-				-	80	0.1	0.01		0.1
				
Total					25.9					1.5

*The prices were obtained as per the rate of supply of commercial grade bulk materials by Hi-Media, India (1 USD = 50 INR).

Process I: Roy Choudhury et.al [8]

Process II: Present study

## Conclusion

In the present study 83.98 g/L pullulan was produced under optimized conditions using DOJSC as nutrient which is significantly higher compared to earlier published reports. Successful use of jatropha oil seed cake as nutrient will help in finding a potential application for this toxic by product of biodiesel industry and also have a significant impact on cost reduction of pullulan production process. It is also to be noted that use of DOJSC as substrate will also help in the process economics for biodiesel produced from jatropha. Thus this will have an advantage of helping both biodiesel and chemical/biochemical industry in terms of waste utilization and cost reduction respectively. In conclusion, the present study suggests a novel utilization for the DOJSC and may be helpful in development of a low cost process for pullulan production. Further studies on process scale up using DOJSC as nutrient are under progress.

## Materials and methods

### Materials

The media components were procured from Hi Media Laboratories (Mumbai, India). Pullulan was purchased from Sigma (St. Louis, USA, Cat. No: P4516:). The de-oiled jatropha seed cake was kindly provided by Dr. Sunil Khare, Indian Institute of Technology (IIT), Delhi, India. It was obtained in bulk (3 Kg), one lot, after production cycle from Biodiesel Production Facility, Centre for Rural Development, IIT-Delhi, India.

### Yeast strain and culture conditions

The yeast strain *Aureobasidium pullulans *RBF 4A3 was isolated from in florescence of *Caseulia axillaries *from places near by Rawatbhata, Rajasthan, India, [8]. The culture was maintained in yeast extract, peptone, dextrose (YPD) agar media (Hi Media, India, Cat. No: MI 363) at 28°C and for long term preservation, 10% glycerol vials were stored at -70°C.

The inoculum was developed by inoculating cultures from fresh YPD agar plates to a 25 ml flask containing 5 ml media composed of 1% yeast extract (Hi Media, India, Cat. No: RM 027), 2% peptone (Hi Media, India, Cat. No: CR 001) and 2% dextrose (Merck, Cat.No: 61780905001730) with subsequent incubation at 28°C for 24 hours at 200 agitation speed. 2.5 ml of the inoculum developed was used to inoculate 50 ml of production media consisting of de-oiled jatropha seed cake (DOJSC) and dextrose in a 250 ml conical flask. The culture flasks were incubated at 28°C and 200 rpm for 120 hours (unless stated otherwise). All the experiments were carried out in triplicate and average values of the results obtained is shown.

### Optimization of culture conditions in shake flask

Single point optimization technique was used to optimize the pullulan production in shake flask level. Factors that have significant effect on pullulan production include concentration of DOJSC, temperature of incubation, agitation speed, inoculum size and initial pH were varied one at a time to obtain optimum conditions. The optimum conditions obtained from each experiment was used in subsequent experiments unless stated otherwise. All the experiments were carried out in triplicate and average of them are reported.

### Effect of concentration of de-oiled seed cake of jatropha in production media

The concentration of DOJSC was varied in the range of 2% (w/v) to 14% (w/v) in the production media and the dextrose concentration was maintained at 15% (w/v) in all cases. The shake flasks were incubated at 28°C and 200 rpm. Samples were withdrawn periodically (at every 24 hour interval) and analyzed for pullulan production, residual sugar content and pH.

### Effect of temperature on pullulan production

The temperature of incubation was varied from 15°C to 30°C. The production media was made of 8% DOJSC and 15% dextrose. All other process conditions were maintained same as mentioned earlier.

### Effect of agitation speed

In the present study, agitation speed was varied from 100 to 350. The flasks were incubated at 28°C and all other conditions remain unchanged.

### Effect of inoculum size

The effect of inoculum size was studied by varying the same from 2% to 10% (v/v) level. The shake flasks were incubated at 200 rpm and all other conditions were maintained same as earlier.

### Effect of initial pH

The initial pH of the medium was varied from 3.5 to 6.5 to study the effect of the same on EPS production. In all cases 5% (v/v) inoculum was used for inoculating the production media and the samples were withdrawn every 24 hours and analyzed for polymer production.

### Analysis, purification and characterization of pullulan

The fermentation broth was centrifuged at 16,000 g for 20 min at 4°C using a Sigma 6 K-15 centrifuge to make it cell free. This cell free broth was subjected to solvent precipitation using 2 volumes of ethanol at 4°C. The precipitate thus obtained was once again separated by centrifugation at 16000 g for 20 min at 4°C. This precipitate was dried at 80°C till constant weight. The pullulan content in the exopolysaccharide was determined by enzymatic method [8]. The pullulan content was expressed in terms of gms of pullulan (dry weight) produced per liter of fermentation broth. The residual sugar content was measured by the Miller's method [32] using a Hitachi U-2900 UV-visible spectrophotometer.

### Validation of shake flask experiments in a laboratory scale fermenter

The optimized conditions obtained in shake flask experiments were validated in a 5 L laboratory scale fermenter (New Brunswick Scientific, Bioflow 310). The production media comprised of 8% (w/v) DOJSC and 15% (w/v) dextrose and it was inoculated with 5% (v/v) inoculum. The fermentation was carried out using 1 vvm air and 350 agitator rpm. The temperature was maintained at 28°C. The process was continued till 120 hours and samples were withdrawn periodically to analyze pullulan production and residual sugar content.

## Competing interests

The authors declare that they have no competing interests.

## Authors' contributions

Roy Choudhury conceived the study, designed the experimentation and drafted the manuscript. Sharma carried out experiments, provided technical inputs and helped in drafting of the manuscript. Prasad supervised the study and corrected the manuscript. All authors read and approved the final manuscript.

## Supplementary Material

Additional file 1 **Figure S1**. FT-IR spectra of standard pullulan (red) and pullulan produced using jatropha as nutrient (black). Absorptions at 3392 cm-1 indicated that both the pullulans have same repeating -OH units as in sugars. Both the samples resemble similarity in the range 1500-650 cm-1, which is characteristic of pullulan. Absorptions at 848 cm-1 and 750 cm-1 indicate the presence of α-D-glucopyranoside units and α-(1-4)-D-glucosidic linkages respectively, whereas, the absorption at 1126 cm-1 indicate the presence of α-(1-6)-D-glucosidic linkages. Click here for file
