# Supplementary material for: Deoiledjatropha seed cake is a useful nutrient for pullulan production
Source: Microb Cell Fact. 2012 Mar 30;11:39. doi: 10.1186/1475-2859-11-39 (PMC3375191; doi:10.1186/1475-2859-11-39)
Supplement: Additional file 1 — Figure S1. FT-IR spectra of standard pullulan (red) and pullulan produced using jatropha as nutrient (black). Absorptions at 3392 cm-1 indicated that both the pullulans have same repeating -OH units as in sugars. Both the samples resemble similarity in the range 1500-650 cm-1, which is characteristic of pullulan. Absorptions at 848 cm-1 and 750 cm-1 indicate the presence of α-D-glucopyranoside units and α-(1-4)-D-glucosidic linkages respectively, whereas, the absorption at 1126 cm-1 indicate the presence of α-(1-6)-D-glucosidic linkages. [file 1475-2859-11-39-S1.DOC]

**Supplementary Figure:**

**FT-IR spectra of standard pullulan (red) and pullulan produced using jatropha as nutrient (black).**

Absorptions at 3392 cm^-1^ indicated that both the pullulans have same repeating –OH units as in sugars. Both the samples resemble similarity in the range 1500-650 cm^-1^, which is characteristic of pullulan. Absorptions at 848cm^-1^ and 750cm^-1^ indicate the presence of α-D-glucopyranoside units and α-(1-4)-D-glucosidic linkages respectively, whereas, the absorption at 1126cm^-1^ indicate the presence of α-(1-6)-D-glucosidic linkages.
